# T Cells Induce Pre-Metastatic Osteolytic Disease and Help Bone Metastases Establishment in a Mouse Model of Metastatic Breast Cancer

**DOI:** 10.1371/journal.pone.0068171

**Published:** 2013-07-18

**Authors:** Ana Carolina Monteiro, Ana Carolina Leal, Triciana Gonçalves-Silva, Ana Carolina T. Mercadante, Fabiola Kestelman, Sacha Braun Chaves, Ricardo Bentes Azevedo, João P. Monteiro, Adriana Bonomo

**Affiliations:** 1 Experimental Medicine Program, Brazilian National Cancer Institute, Rio de Janeiro, Brazil; 2 HCIII, Brazilian National Cancer Institute, Rio de Janeiro, Brazil; 3 University of Brasília, Institute of Biology, Brasilia, Brazil; 4 Lymphocyte Biology Section, Laboratory of Systems Biology/NIAID/NIH, Bethesda, Maryland, United States of America; 5 Laboratory on Thymus Research, Oswaldo Cruz Institute, FIOCRUZ, Rio de Janeiro, Brazil; 6 Immunology Department, Microbiology Institute Prof. Paulo de Góes, Federal University of Rio de Janeiro, Rio de Janeiro, Brazil; Shanghai Jiao Tong University School of Medicine, China

## Abstract

Bone metastases, present in 70% of patients with metastatic breast cancer, lead to skeletal disease, fractures and intense pain, which are all believed to be mediated by tumor cells. Engraftment of tumor cells is supposed to be preceded by changes in the target tissue to create a permissive microenvironment, the pre-metastatic niche, for the establishment of the metastatic foci. In bone metastatic niche, metastatic cells stimulate bone consumption resulting in the release of growth factors that feed the tumor, establishing a vicious cycle between the bone remodeling system and the tumor itself. Yet, how the pre-metastatic niches arise in the bone tissue remains unclear. Here we show that tumor-specific T cells induce osteolytic bone disease before bone colonization. T cells pro-metastatic activity correlate with a pro-osteoclastogenic cytokine profile, including RANKL, a master regulator of osteoclastogenesis. In vivo inhibition of RANKL from tumor-specific T cells completely blocks bone loss and metastasis. Our results unveil an unexpected role for RANKL-derived from T cells in setting the pre-metastatic niche and promoting tumor spread. We believe this information can bring new possibilities for the development of prognostic and therapeutic tools based on modulation of T cell activity for prevention and treatment of bone metastasis.

## Introduction

The role of the immune system in controlling cancer was first hypothesized more than one hundred years ago [1]. However, the concept of Immunosurveillance as a response of the adaptive immune system came up with the proposition of the Clonal Selection Theory by Burnet and the demonstration that tumor specific antigens in fact exist [1,2]. More recently, immune selection of malignant cells based on differences on antigen specificities supported the idea of “immunoediting” [1,3,4] adding the possibility of a pro-tumoral activity to the previously proposed concept of immunosurveillance. Once the tumor is “shaped” by the immunoselection mechanisms, it will be in equilibrium with the host immune system, until it can escape. To escape, a tumor cell must modify its intrinsic and extrinsic factors [5,6], favoring its own growth. In fact, extrinisic factors represented by stromal cells, extracellular matrix and hematopoietic cells [7–10] can be either protective or pro-tumorigenic.

Regarding the immune system, tumor cells might express co-inhibitory molecules and secrete cytokines that will subvert the immune response [1,5,11]. Tumor associated macrophages (TAM), for example, characterized as M2 subtype, can produce a series of cytokines that will favor tumor growth and lung metastasis [12,13] in response to Th2 cells modulation [14]. When it comes to bone metastasis, although the role of osteoclasts (a specialized bone macrophage) in creating a permissive environment for tumor colonization is well known [15,16], the role of T cell in regulating osteoclasts in bone metastasis and cancer induced bone disease is not known [17,18]

The presence of T cells in the bone cavity has been well documented. Bone marrow CD4^+^ T cells are involved in the control of normal hematopoiesis [19] and are present in the hematopoietic stem cell niche [20], which is also occupied by cancer metastasis [21]. As an active component of the bone marrow microenvironment [22], CD4^+^ T cells have also been found to have an impact on the bone remodeling process through induction or regulation of molecules, such as RANKL, involved in bone metabolism [23–25]. RANKL, is a pleiotropic molecule expressed by different cell types and with multiple functions [26,27]. In bone tissue physiology, RANKL is a key molecule which promotes osteoclast (OC) differentiation and activation, and its absence in osteoblasts, chondrocytes or osteocytes leads to abnormal bone formation or remodeling [28,29]. RANKL is also present in CD4^+^ T cells after activation [27] and it was shown to be preferentially expressed in Th17 cells [30]. Although, these cells are clearly involved in the pathogenesis of autoimmune arthritis, and are therapeutic targets in both experimental and human disease [31,32], no direct role of Th17 cells in bone loss has been shown until now. Th17 cells have been shown to induce osteoclastogenesis indirectly, through induction of RANKL expression in osteoblasts and synoviocytes [30].

Since T cells can “shape” the tumor, orchestrate metastatic colonization to the lungs, and are active components of the inflammatory osteolytic disease, it seemed reasonable to ask if T cells from mice bearing a bone metastatic tumor would play any role in the osteolytic bone disease and/or bone and BM colonization.

## Material and Methods

### Detection of primary tumor growth and spontaneous metastasis

All animal experiments were in accordance to the Brazilian National Cancer Institute (INCA) guidelines for animal use in research and approved at CCS animal committee at Federal University of Rio de Janeiro (license number IMPPG027). Females BALB/c and BALB/c nude mice were obtained from INCA or IPEN/CNEN/USP. The tumor lines 67NR and 4T1 were kindly provided by Dr. Fred Miller from Karmanos Cancer Institute, Detroit, MI [33]. Female BALB/c mice (6-8 weeks old) were inoculated with 10^4^ cells in the fourth mammary fat pad. Primary tumors maximum diameter was obtained by ultrasound measurement [34]. Numbers of metastatic cells in LNs and indicated bones were determined using a clonogenic metastatic assay supplemented with 6-thioguanine. Presence of metastatic cells was also evaluated by RT-PCR to cytokeratin 19 (CK19), and GAPDH for normalization. To prepare 4T1 soluble tumor-Ag (sAg), tumor, were dissected, ressuspended in ice cold PBS, filtered through 40 µm cell strainer, disrupted by freezing and thawing 5x, boiled for 10 min and centrifuged at 14,000 rpm, for 30 min, at 4^°^C.

### In vitro assays for osteoclast formation and activity

Freshly isolated femur BM cells from BALB/c mice (6–8 weeks old) were cultured at a density of 1x10^5^ cells per well, in 24-well plates, in DMEM plus 10% FBS, containing supernatants from sAg stimulated iliac BM cells in the presence of M-CSF (10ng/mL), with or without recombinant OPG (10ng/mL) (Peprotech) or rat anti-mouse IL-17F mAb (10ng/mL) (R&D systems), for 7 days, at 37^°^C. Positive controls received recombinant RANKL (10ng/mL) (Peprotech). TRAP staining (Sigma) and pit formation assays (osteologic disks from BD Biosciences) were carried according to the manufacturer’s protocol. TRAP-positive cells containing three or more nuclei were counted as OCs.

### Analysis of serum cytokine and production by LN, spleen or BM derived T cells

Single cell suspensions from bones were obtained after collagenase Type I (1 mg/mL) and DNase (100 µg/mL) treatment at 37^°^C, for 60 min. After mechanical disruption, draining LNs, spleens, and the indicated bones were cultured (10^7^ cells/ml) with 50µg/mL of sAg, in 24-well plates for 72 hs. Cytokine content was measured by ELISA (R&D Systems). Flow cytometry was performed 3 days after sAgstimulation. PMA (20 ng/mL, Calbiochem) and ionomycin (0.2 µg/mL, Sigma-Aldrich) were added to the last 4 hs and, brefeldin A (Sigma-Aldrich) for the last 2 hs of culture. Anti-mouse CD16/32 mAb (clone 2.4G2) was used for Fc blockage. PE-Cy5-conjugated rat anti-mouse CD3 mAb (clone 145-2C11), APC-conjugated rat anti-mouse CD4 mAb (clone GK 1.5), FITC-conjugated rat anti-mouse IL-17F mAb (clone 316016), PE-conjugated rat anti-mouse RANKL mAb (88227), or isotype controls (BD Biosciences) were used and data collected on a FACSCalibur® (BD Biosciences) and analyzed using FlowJo® software (Tree Star).

### Bone Histomorphometry and Micro-Computed Tomography

 Iliac bones from BALB/c or nude mice transferred with T cells were fixed in 10% formalin, decalcified in 20% of EDTA for two weeks, and embedded in paraffin. 5µm serial sections were stained with H&E or TRAP according to standard techniques. Slides were scanned using scanscope (A p e r i o®). Bone histomorphometry was performed using a semiautomatic image analysis program (Motic®). TRAP-positive stained OCs were assessed in the same tissue sections and expressed as number of OCs/mm of bone length. Iliac bones were also fixed in 70% ethanol and high resolution microtomography nondestructive three-dimensional evaluation of bone volume. Bones were scanned in Skyscan® 1076 MicroCT (Skyscan, Kontich, Belgium) at 70 kV, 141 µA, Al 0.5 mm filter and 12.56 pixel size. Reconstruction was performed using Nrecon software (Skyscan, Kontich, Belgium), using for smoothing, beam-hardening and ring-artifact, correction respectively 1, 30 and 10 levels. Grey scale range was set from 0.0000 to 0.0411 HU. The reconstructed MicroCT files were used to analyze the samples and to create volume renderings of the region of interest. Bone volume and mineral density was performed using CTAnalyser software (Skyscan, Kontich, Belgium).

### Adoptive transfers

Briefly, 11 days after tumor inoculation, bone marrow cells were obtained as described above. T cells were positively selected using magnetic beads covered with anti-mouse CD3 (Miltenyi Biotec). CD3^+^ purified T cells (more than 90% pure) were adoptively transferred (1x10^6^ cells/mouse), i.v., into naïve female BALB/c nude mice (6 mice/group) along with single dose of sAg (25 µg/mouse). In another experimental set, total LN cells from naïve or tumor-bearing BALB/c mice were used, and 67NR tumor cell were injected into the mammary fat-pad as the Ag source. Two weeks later, splenocytes were stimulated *in vitro* with sAg (50µg/mL) or rat anti-mouse CD3 (1µg/mL). Non-stimulated cells from all groups were used as controls. Cells were analyzed by flow cytometry and supernatants were evaluated by ELISA, as previously described.

### RANKL and IL-17F knock-down in T cells of 4T1-tumor bearing mice and mRNA evaluation of CD3^+^ cells.

In order to knock-down RANKL and IL-17F in LN T cells of 4T1 tumor-bearing mice, cells were transfected with specific murine shRNA (RANKL shRNA Plasmid (m): sc-37270-SH and IL-17F shRNA Plasmid (m): sc-146204-SH, SantaCruz Biotechnologies) using AMAXA transfection kit for primary murine T cells (VPA-1006, Amaxa® Mouse T Cell Nucleofector® Kit, Lonza). Final concentrations of plasmids were 3 µg, or 6 µg for double transfection. 3 hs after transfection, viable T cells (50–60%) were adoptively transferred into BALB/c nude mice along with sAg (25 µg/mouse). The presence of injected cells in spleens and BMs of nude mice was examined in the end of experiments (day 6 after transfer) by RT-PCR using mouse specific primers to CD3 and GAPDH for normalization.

### Statistical analyses

Data values are expressed as the mean±SD, from at least three independent experiments. Statistical differences between mean values were evaluated by ANOVA, and pairwise comparisons were done by the Tukey test. *p* values of ≤ 0.05 or ≤ 0.001 were considered to be statistically significant (minimum n=3). Arabic letters indicate significant differences amongst groups.

## Results

### Animals bearing breast metastatic tumors produce high levels of pro-osteoclastogenic cytokines

**Figure 1:**
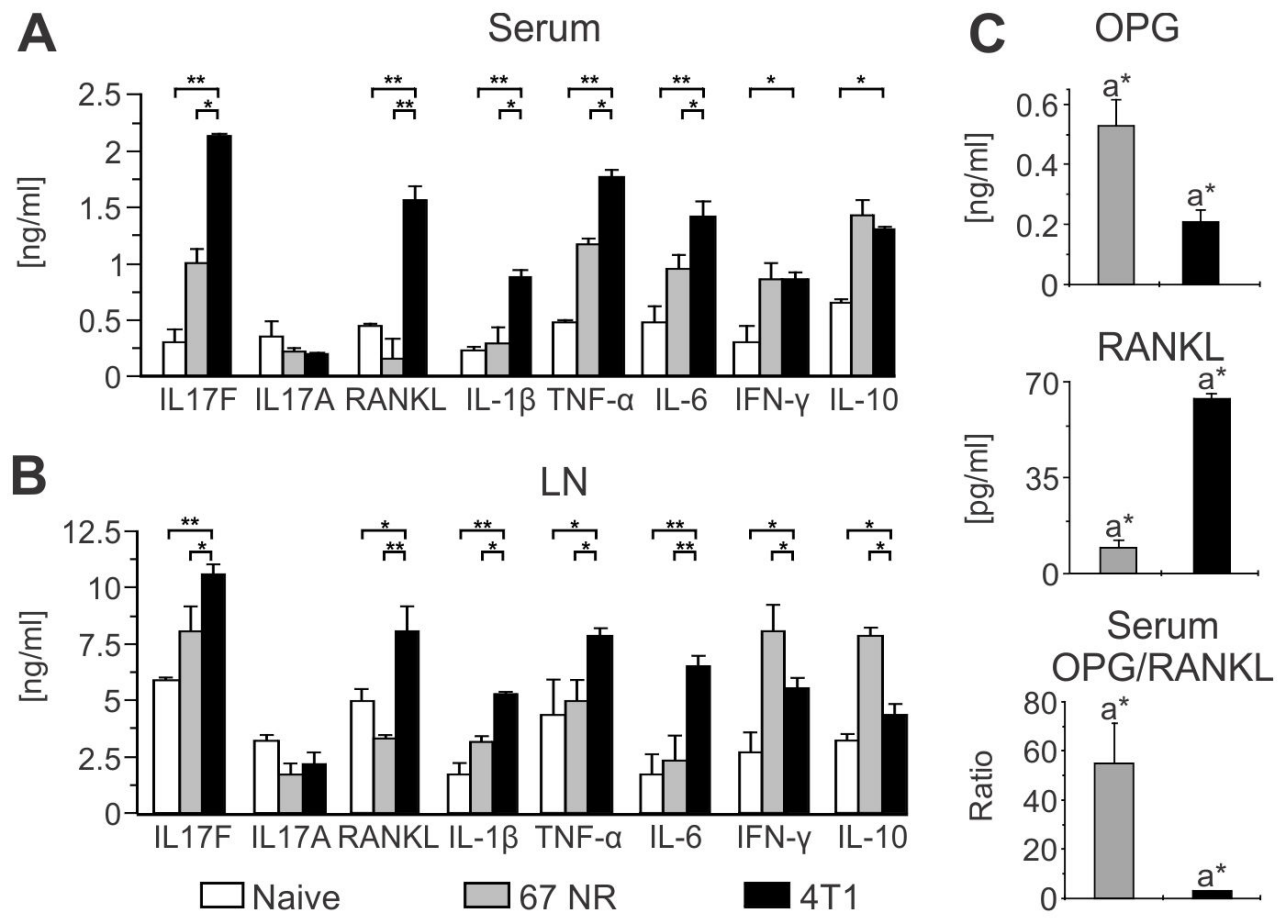
Metastatic 4T1 tumor stimulates the production of pro-osteoclastogenic cytokines. (**A**–**B**) BALB/c female mice were orthotopically injected in the mammary fat pad with 10^4^ metastatic 4T1 or non-metastatic 67NR tumor cells. Cytokine production was evaluated by ELISA in the sera (**A**) and supernatants of αCD3-stimulated cells (**B**) collected from inguinal draining LNs 35 days post tumor injection. Naïve animals were used as negative controls. Data are expressed as the mean ± SD of five mice/group and are representative of at least three independent experiments. *p<0.05; **p<0.001. (**C**) Serum concentration of OPG and RANKL, and the OPG/RANKL ratio in tumor-bearing BALB/c mice, measured by ELISA, 35 days after injection of 4T1 or 67NR tumor cells. a*p≤0.05. Data are expressed as the mean ± SD of five mice/group.

In order to examine whether there was a relationship between tumor invasiveness and a specific pattern of immune response, we used as a model two sibling cell lines, derived from a spontaneous mammary gland tumor from a BALB/c mouse [35]. The 67NR cell line presents a local and self-contained growth, while its sibling 4T1 shows an invasive behavior with development of metastases to the LN, bones and lungs among other tissues. Female BALB/c mice were implanted in the fourth mammary fat pad either with 4T1 or 67NR cell lines, and 35 d after tumor injection, we compared the cytokine levels in the serum and in the supernatants of anti-CD3 stimulated LN cells from mice of both groups (Figure 1, A and B). Significantly higher levels of the pro-osteoclastogenic cytokines IL-17F, RANKL, IL-1β, TNFα, and IL-6 were detected in the sera and supernatants from LN-stimulated cells of animals bearing the metastatic tumors than in mice with non-metastatic tumors. Conversely, the levels of anti-osteoclastogenic cytokines such as IFN-γ [25,36] and IL-10 [37] were higher in the group of mice implanted with non-metastatic tumors. Moreover, the ratio between OPG (a decoy receptor for RANKL) and RANKL was 10 to 20 fold lower in the serum of animals bearing metastatic tumors than in mice with non-metastatic tumors. The low OPG/RANKL ratio happened at the expenses of both a decreased OPG and an increased RANKL level, surely indicating a pro-osteoclastogenic activity (Figure 1C). These results show that a prominent pro-osteoclastogenic cytokine profile is present in animals bearing 4T1 metastatic, but not 67NR non-metastatic tumors.

### Bone marrow pro-osteoclastogenic cytokine production in response to tumor-antigen stimulation precedes metastatic colonization of the bone marrow

**Figure 2:**
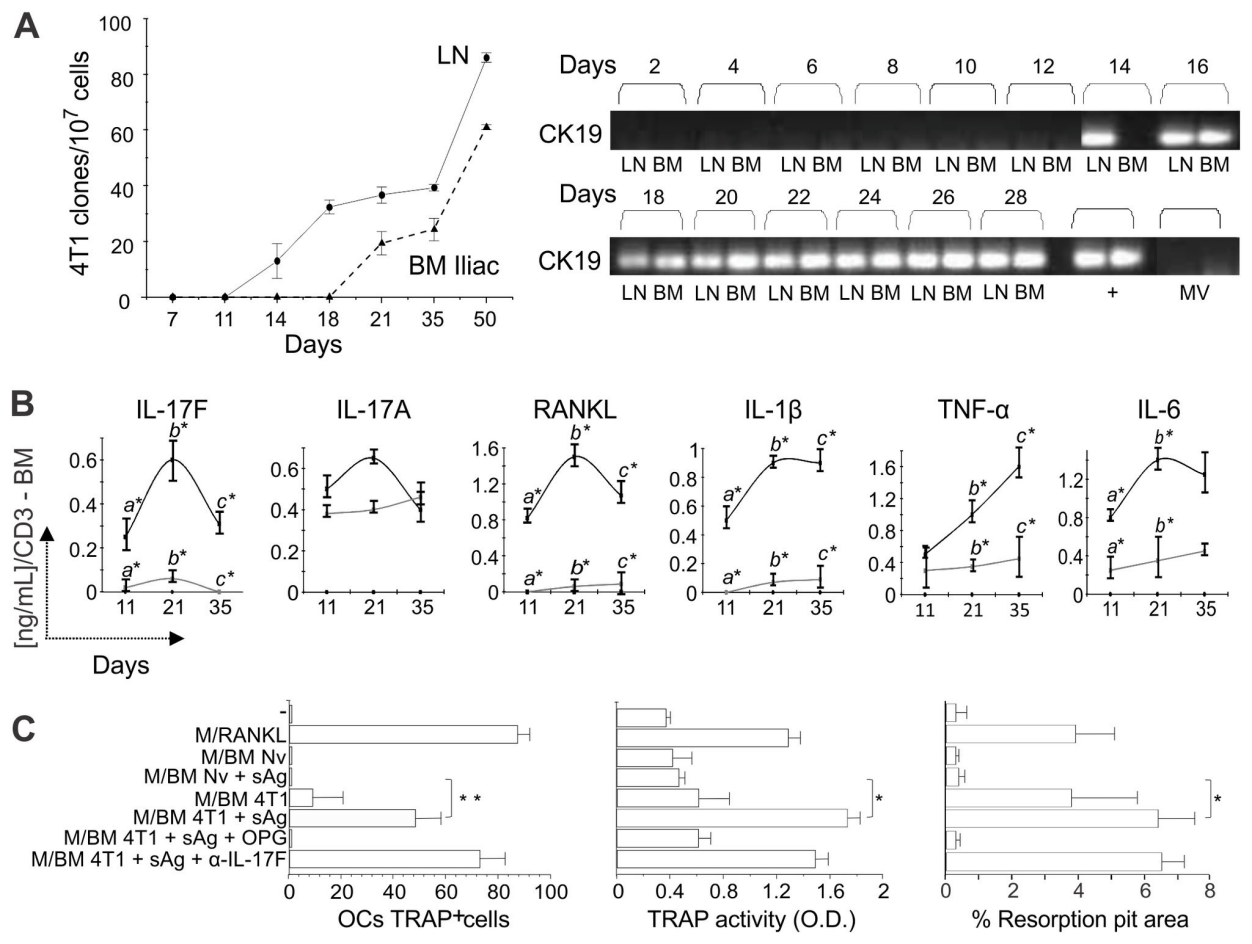
Pro-osteoclastogenic cytokine production by BM cells in response to tumor antigenic stimulation precedes metastatic colonization of the bones. (**A**) Clonogenic metastatic assays were performed with cells obtained from the draining LNs and iliac BM of BALB/c mice orthotopically injected with 10^4^ 4T1 tumor cells. The number of metastatic clones was determined at different time points, using a 6-thioguanine resistant assay (left panel). Expression of cytokeratin 19 (CK19) was also determined in such samples by RT-PCR (right panel). 67NR or 4T1 tumor derived microvesicles (MV) were used as a control to ascertain that a positive RT-PCR indicates exclusively the presence of whole tumor cells in the target organ. 4T1 cells mRNA was obtained from *in vitro* cultures or *in vivo* tumors (+) and was used as a positive control in the RT-PCR assay. (**B**) Kinetics of pro-osteoclastogenic cytokine production in response to tumor soluble antigen (sAg) stimulation of BM cells obtained from iliac bones of mice bearing 4T1 or 67NR tumors. Naïve animals were used as controls. Supernatants of sAg stimulated iliac BM cells were harvested after 72hs and cytokine production was measured by ELISA. Data are expressed as the mean ± SD of five mice/group and are representative of at least three independent experiments. *p<0.*05*. (**C**) Osteoclastogenesis assays using naïve BM cells cultured in the presence of either recombinant M-CSF and RANKL, or M-CSF and supernatant of iliac BM cells derived from 4T1 tumor-bearing mice (11 d after tumor injection). Supernatant from iliac BM cells of naïve animals (BM Nv) stimulated or not with sAg was used as specific control. Cultures were also treated with r-OPG or α-IL17F as indicated. The number of TRAP^+^ multinucleated OC cells obtained *in vitro* was determined (left panel) and TRAP activity in such supernatants was measured by a colorimetric assay (middle panel). In the right panel, generation of functional OC cells in vitro was also determined using BD BioCoatTM OsteologicTM Bone Cell Culture System (BD Biosciences). The graphic represents the resorbed area on osteologic discs. All data are from at least two independent experiments (n=5 mice/group) and presented as mean ± SD. *p<0.05; **p<0.001.

To understand if the production of pro-osteoclastogenic cytokines observed at 35 d after tumor implantation is cause or consequence of metastases to the bone, we looked at the kinetics of the colonization of the bone cavity by tumor cells as well as the kinetics of production of pro-osteoclastogenic cytokines in the marrow microenvironment. Using a clonogenic metastatic assay as well as molecular analyses, we observed that no metastatic clones are present in the draining LNs of the tumor until day 14 p.i., and until day 16 p.i. in the BM (Figure 2A).

We then looked at the profile of cytokine production in the marrow microenvironment. To do that, BM cells from animals bearing 4T1 or 67NR tumors were collected from day 11 to d 35 p.i., the cells were stimulated with tumor soluble antigen (sAg) in vitro and level of different cytokines was measured by ELISA (Figure 2B). We found that bone marrow cells from metastatic 4T1 bearing mice secreted higher concentrations of pro-osteoclastogenic cytokines than cells from 67NR bearing animals, even at early time points. Of note is the fact that BM cells from naïve animals do not produce any detectable cytokines after sAg stimulation. This pattern was observed in all bones tested (Figure S1A), although it was more prominently seen in the iliac bone, which is rich in trabecular bone and also a major site of metastasis (Figure S1B). Surprisingly, we observed no differences in the expression of IL-17A – a T cell–derived cytokine that is involved in the pathogenesis of osteolytic lesions in rheumatoid arthritis [30,31] – between BM cells from mice bearing metastatic or non-metastatic tumors (Figure 2B). Increased levels of IL-17F were observed in response to tumor antigens nonetheless.

To ascertain that the T cell pro-osteoclastogenic phenotype observed in the BM could lead to generation of functional OCs, we generated supernatants from tumor-stimulated BM cells, and tested the ability of these supernatants to induce osteoclastogenesis in BM cell cultures in vitro, in the presence of M-CSF, a cytokine required to induce RANK expression in the marrow pre-OCs. Supernatants from T cells stimulated with 4T1 antigens induced functional OCs differentiation (Figure 2C, left panel). This was confirmed when TRAP activity was measured (Figure 2C, middle panels). These differentiated cells were competent as they consumed mineral matrix present in osteologic disks in vitro (Figure 2C, right panel). These functional activities were inhibited by OPG, a decoy receptor for RANKL, but not by anti-IL-17F, suggesting that T cell derived RANKL is the major osteoclastogenic molecule in this setting.

### Bone loss precedes metastatic colonization of the bone cavity

**Figure 3:**
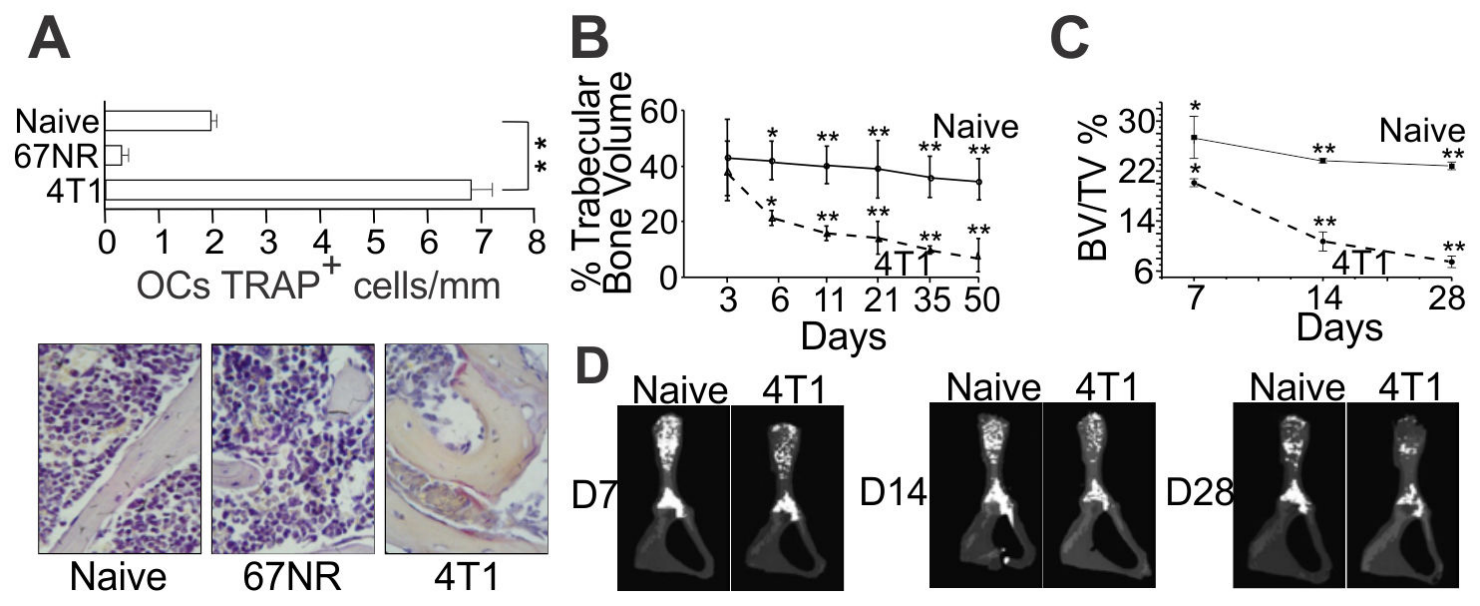
Increased number of osteoclasts in 4T1 tumor-bearing mice relates to early bone loss. (**A**) Number of TRAP^+^ multinucleated OC cells was determined in iliac bones, 11 days after 4T1 or 67NR tumor cells injection in mammary fat pad. Representative TRAP-stained sections are shown (original magnifications 40x). (**B**) Histomorphometric analysis of iliac bones from naïve and 4T1 tumor-bearing mice, at different time points after tumor injection in the mammary fat pad. Sagital sections from demineralized iliac bones were made following conventional methods and stained with H and E. All microscopic slides were scanned with a ScanScope GL equipped with a 40x objective. Trabecular bone volume was expressed as a percentage of total tissue volume. (**C**–**D**) High resolution µCT analysis. BV/TV%, trabecular bone volume/tissue volume were calculated from µCT images. Results are expressed as mean ± SD and are representative of at least three independent experiments with 5 mice/group. ^*^ *p<0.05*; ^**^ *p<0*.*001*.

To understand the impact of pro-osteoclastogenic cytokines production in the bone dynamics, we looked at its effect on osteoclastogenesis and bone mass in vivo. We evaluated osteoclastogenesis by counting the number of multinucleated TRAP^+^ cells per millimeter (mm) of bone surface in the different conditions. We found a large increase in the number of OCs in mice implanted with metastatic 4T1 cells as compared to naïve mice or animals bearing 67NR non-metastatic tumor cell (Figure 3A). This increase already is evident on day 11 post tumor implant, when bone metastasis is still absent. More important, not only an increased osteoclastogenesis was observed but also a rapid and early bone loss in 4T1 tumor bearing mice was evident by histomorphometry and µCT (Figure 3, B–D). Indeed, by day 6 p.i., almost 50% of trabecular bone had been resorbed. The above results show that significant bone loss precedes bone metastatic colonization in animals bearing 4T1 tumor cells.

### T cells are required for development of pre-metastatic osteolytic disease

Since metastases to the bone cavity were not found before day 16 after tumor injection and therefore cannot be responsible for the early bone loss observed, we asked whether that was actually the result of T cell pro-osteoclastogenic activity.

First we looked at T cell numbers in the draining LN and bone marrow, starting at day 11 after tumor inoculation. Although, by day 11, the relative numbers of CD3+, CD4+ and CD8+ were the same in animals bearing 4T1 or 67NR tumor cells, the absolute numbers of CD4+, but not CD8+ T cells in the BM of 4T1 positive mice were already higher than in the other groups (Figures S2A and S2B). Since these tumors are derived from BALB/c mice and MMTV positive, we checked whether this early increase in CD4 T cell numbers in the BM could be the result of superantigen stimulation. No TCRVβ skew was observed in the BM or LN of 4T1 bearing mice when compared to naïve animals (Figure S2C) indicating that no detectable superantigen stimulation is taking place.

**Figure 4:**
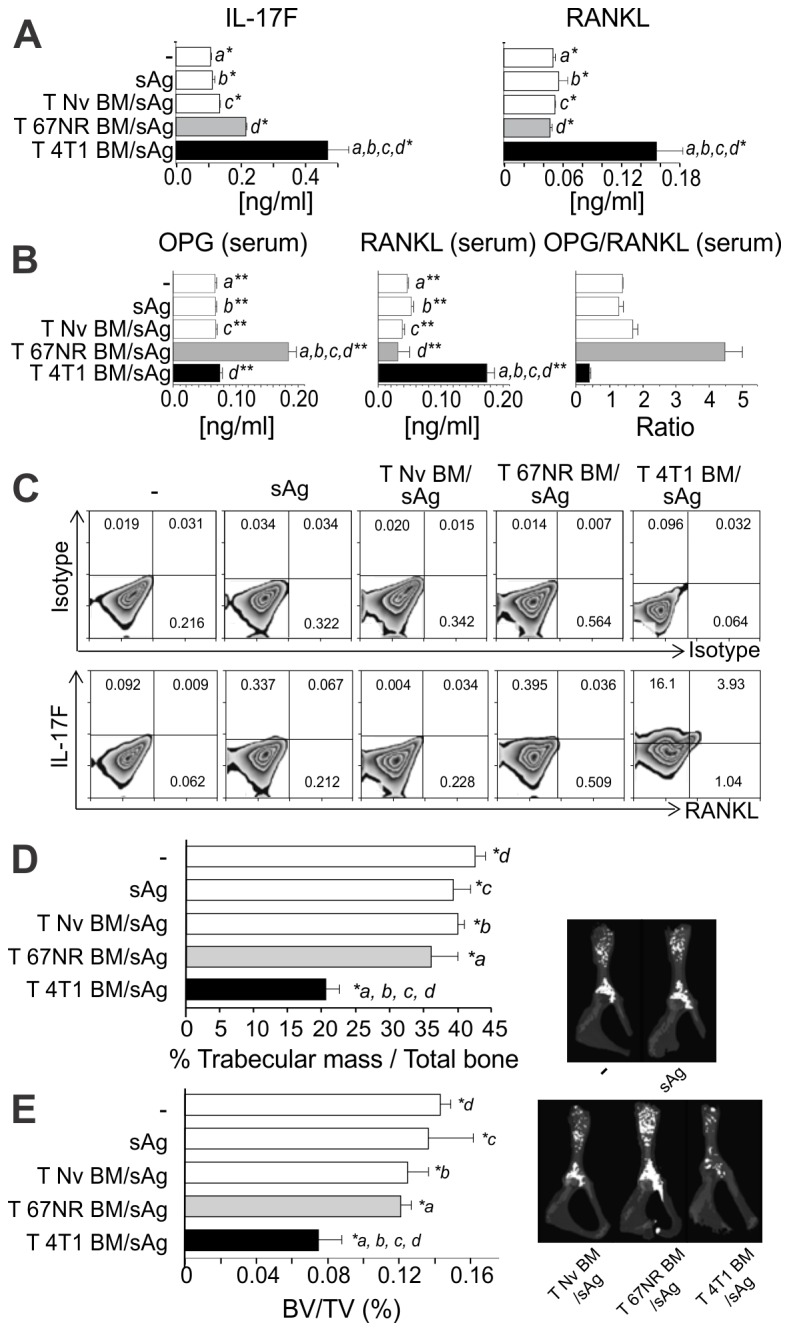
Early bone loss in 4T1 tumor-bearing mice is T cell mediated and **independent of metastatic colonization.** CD3^+^ T cells derived from iliac BM of BALB/c mice, 11 days after 4T1 (T 4T1) or 67NR(T 67NR) tumor cells injection into the mammary fat pad, or control T cells from naïve mice (T Nv) were transferred intravenously to athymic nude mice and challenged with the soluble fraction of tumor antigen lysate (sAg). (**A**) 14 days after transference, spleen cells were restimulated with sAg and IL-17F and RANKL production was evaluated by ELISA. Data are expressed as the mean ± SD of five mice/group and are representative of two independent experiments ^*^ *p≤0.05.* (**B**) Frequency of IL-17F^+^ RANKL^+^ T cells was assessed by flow cytometry, 14 days after T cells transference. Plots show data from CD3^+^ CD4^+^ gated T cells. (**C**) Serum concentrations of OPG and RANKL and the OPG/RANKL ratio, measured by ELISA, 14 days after T cells transference. Data are expressed as the mean ± SD of five mice/group. a, b, c ,d** p<0.001. (**D**) Histomorphometric analysis of the iliac bones from mice of the different groups and (**E**) high resolution µCT analysis of the iliac bones. Both analyses were performed as described in Figure 3. Results shown are representative of at least two independent experiments with 5 mice/group. a, b, c ,d* p<0.05.

To test if T cells were indeed responsible for the early bone loss observed, CD3^+^ T cells were purified from the BM of 4T1 or 67NR bearing BALB/c donor mice 11 days after tumor inoculation (one week before detection of bone cavity metastases - Figure 2A) and i.v. transferred to T cell-deficient BALB/c Nude (nude) recipients along with 4T1 sAg. After 14 days, splenocytes from recipient mice of the different groups were restimulated in vitro with sAg for 72h. Supernatants obtained from the cultures were harvested and RANKL and IL-17F levels were measured by ELISA. Production of IL-17F and RANKL was observed only in supernatant obtained from cells derived from donor mice bearing 4T1, but not 67NR, tumors (Figure 4A). In line with these results, flow cytometric analyses showed the presence of IL-17F ^+^ RANKL^+^ CD4 T cells in the spleen of nude mice that received cells from 4T1-bearing donors (Figure 4B); On the other hand, IL-17F ^+^ RANKL^+^ double positive cells were absent in the CD8+ population (Figure S4B). Also, analysis of the serum showed a low OPG/RANKL ratio (Figure 4C). Altogether, these results indicate that the T cell cytokine profile observed in the bone marrow of 4T1 and 67NR bearing mice is preserved after transfer of BM T cells to *nude* mice and is not dependent on the presence of live tumor cells.

Importantly, bone histomorphometry and µCT analyses showed that T cells derived from 4T1-bearing donors were capable of inducing bone loss in the presence of tumor antigens but in the absence of tumor cells (Figure 4, D and E). Of note is the fact that very early after T cell transfer, by day 6, bone loss was already evident no matter what microCT parameters were analysed (Figure S3). Similar results were obtained when pre-metastatic LN T cells were transferred into *nude* mice (Figure S4). In this case, the source of antigen was the 67NR cell line indicating that both, 4T1 e 67NR share the specific epitopes recognized by T cells.

### T cell-induced pro-osteoclastogenic activity is dependent on RANKL expression by T cells

**Figure 5:**
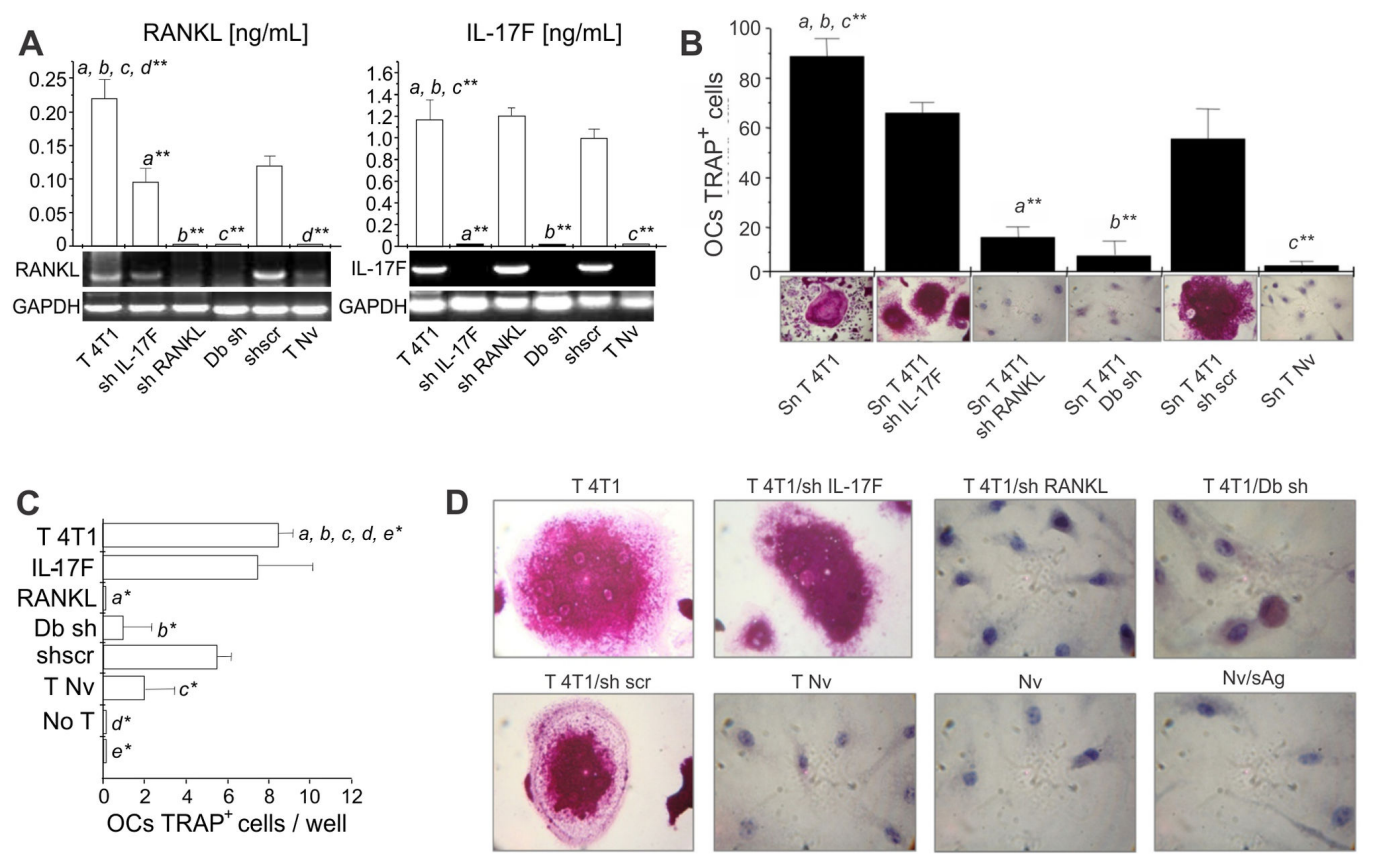
Specific blockage of RANKL expression, but not IL17F, in T cells abolishes the *in* *vitro* pro-osteoclastogenic activity of tumor-specific T cells. LN cells obtained from 4T1 tumor-bearing BALB/c mice (T 4T1), 11 days after tumor injection, were transfected with shRNA for IL17F (shIL17-F), RANKL (shRANKL), scramble (scr), or both RANKL and IL17F (Db sh). These cells were transferred intravenously to athymic nude mice and the recipients were challenged with soluble tumor antigen (sAg). (**A**) Knocked down cells were re-stimulated *in vitro* with sAg, and assayed for RANKL and IL17 expression by ELISA or RT-PCR, prior to injection into recipient mice. ^**^ *p<0.001*. (**B**) The osteoclatogenic activity of supernatants obtained from knocked down cells, stimulated *in vitro* with sAg for 3 days, was also evaluated in osteoclastogenic assays (as described in Figure 2C). The number of TRAP^+^ multinucleated OCs was determined per well and the representative TRAP staining of OCs is shown under each graphic bar. (**C**–**D**) Spleen cells recovered 6 days after i. v. transfer were restimulated *in vitro* with sAg and the osteoclastogenic activity of the supernatants obtained was also tested over naive BM cells. The number of TRAP^+^ multinucleated OCs was determined per well and the representative TRAP staining of OCs is shown in panel D ^*^ *p≤0*.*05*.

To understand the mechanisms involved in the pre-metastatic bone loss mediated by T cells we studied how the inhibition of IL17-F and RANKL expression in T cells would affect osteoclastogenesis. T cells were collected from LNs of 4T1 tumor bearing mice and IL17F or RANKL expression was suppressed using specific shRNA (Figure 5A). Silenced T cells were stimulated in vitro, their supernatants were harvested and tested for pro-osteoclastogenic potential using an in vitro assay. Silencing RANKL, but not IL-17F, indeed impairs osteoclastogenesis, indicating that T-cell derived RANKL plays a major role in the process (Figure 5B). Silenced T cells were also transferred to nude recipients along with tumor antigen. After 6 days post i.v. transfer, the phenotype of the transferred T cells still was maintained as shown by an in vitro osteoclastogenic assay using supernatant derived from in vitro stimulated spleen cells (Figure 5, C and D). Again, osteoclastogenesis was observed only in the presence of RANKL confirming our previous results.

### Pre-metastatic osteolytic disease requires RANKL expression by T cells

**Figure 6:**
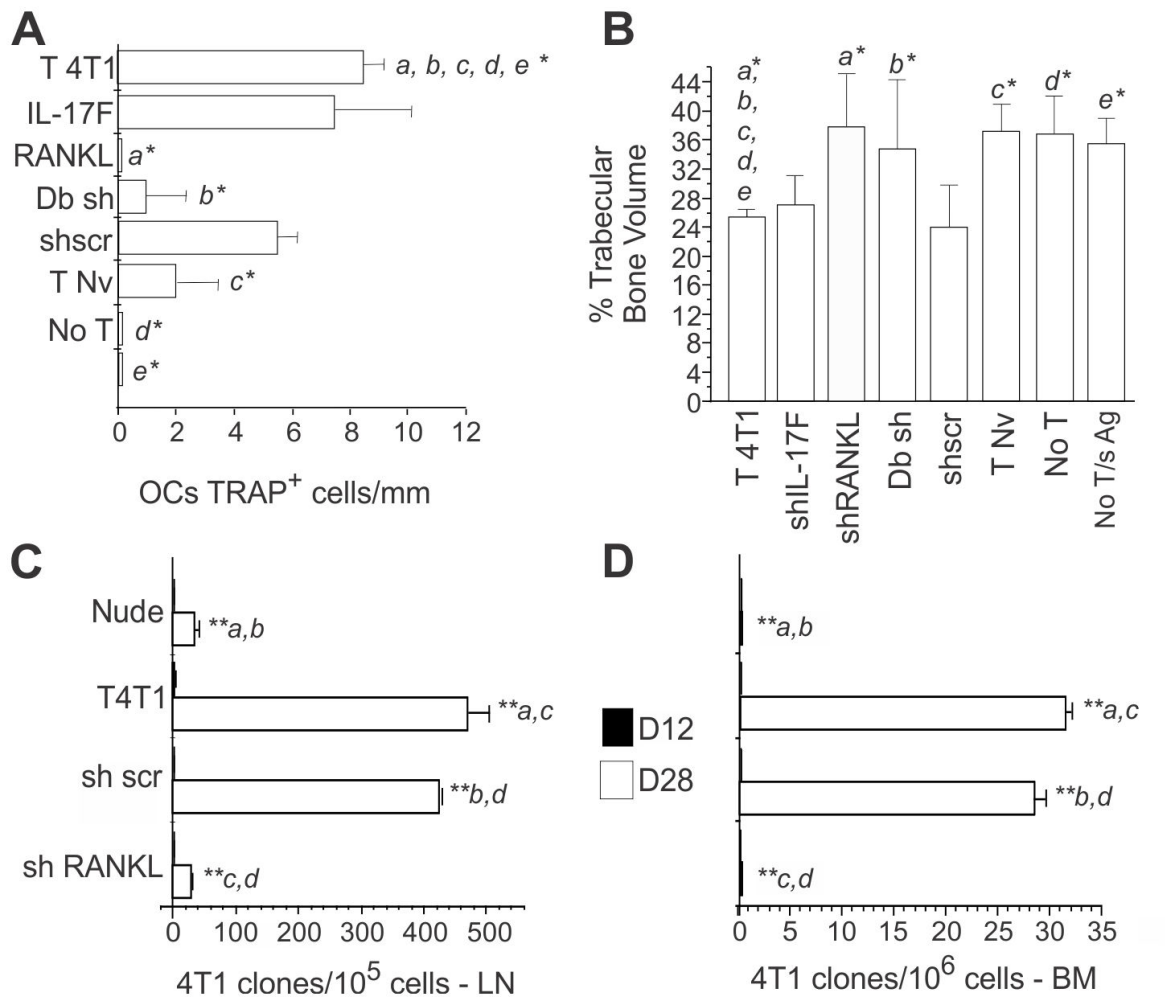
T cell-induced osteoclastogenesis and bone loss requires expression of RANKL in T cells which “helps” metastatic colonization (A) LN cells obtained from 4T1 tumor-bearing BALB/c mice (T 4T1), 11 days after tumor injection, were transfected with shRNA for IL17F (shIL17-F), RANKL (shRANKL), scramble (scr), or both, RANKL and IL17F (Db sh), transferred i. v. into athymic nude mice and challenged with soluble tumor antigen (sAg). Bone sections from recipient mice were prepared and the number of TRAP^+^ OCs/mm of bone surface was determined. (B) High resolution µCT of iliac bones from the different groups of nude mice transferred with the indicated T cells. Results shown are representative of two experiments with 5 mice/group). ^*^ *p*≤0.05; ^**^ *p≤0.001*. (C–D) Number of metastatic clones in the LNs and iliac BMs was assessed by clonogenic metastatic assay in the recipient mice on day 12 and 28. Nude, non-reconstituted control; T 4T1; reconstitution with 4T1 T cells; sh scr, sh Scramble T 4T1; sh RANKL, sh RANKLT 4T1. Results shown are representative of two experiments with 6 mice/group). ^**^ *p≤0*.*001*.

Next, we evaluated if inhibition of RANKL expression in the T cells from 4T1 bearing mice (4T1 T cells) also has an impact in bone loss. RANKL knocked down T cells were transferred to nude recipients and the number of OCs present in the endosteal surface was evaluated in vivo. We found that an increase in the number of OCs was observed in nude mice receiving 4T1 T cells (4T1 T) when compared to nude recipients that did not receive 4T1 T cells and/or antigen (T Nv, sAg and No T/sAg groups) (Figure 6A and Figure S5A). However, this increase was not observed if the transferred 4T1 T cells were unable to produce RANKL. Moreover, pro-osteoclastogenic activity does not depend on the production of IL-17F (Figure 6A and Figure S5A). Bone loss is indeed observed in the absence of IL-17F but is inhibited by the absence of T cell-derived RANKL (Figure 6B and Figure S5B and C).

### T cell-induced pre-metastatic osteolytic bone disease is required for metastatic colonization of the bone cavity

Our results show that T cell-derived RANKL is necessary for the induction of a pre-metastatic osteolytic disease. If osteolytic disease is a requirement for tumor establishment or bone metastasis initiation, as predicted from the vicious cycle hypothesis [15], we should be able to interfere with the bone metastatic process by inhibiting RANKL production by T cells. To test this hypothesis, RANKL expression was knocked down in 4T1T cells, and these cells were transferred to Nude recipients that were also implanted with 4T1 tumor cells in the mammary fat pad. While the primary tumor growth was only delayed in mice that did not receive any T cells or mice receiving RANKL silenced T cells (Figure S6A), the effects of RANKL knock down in lymph node and bone metastases were striking. In the absence of T cell-derived RANKL, metastasis to the LN was reduced to 5% of what was observed in the positive control group (T 4T1), while development of bone metastases was completely inhibited (Figure 6, C and D). Accordingly, bone metastases were also absent, or present in very small number (until day 28), in nude recipients that were not reconstituted with T cells (Figure 6D). This is not a consequence of a diminished primary tumor growth since metastasis to the lungs are increased in the absence of T cell derived RANKL (Figure S6B).

These results indicate that RANKL^+^ T cells provide help for bone, but not to lung metastasis establishment unveiling an unexpected role of T cells in promoting bone tumor spread.

## Discussion

Although immune activity is classically linked to anti-tumor activity several reports were published in the past linking immunity to tumor progression [38–40]. We show here that indeed this can be the case. Using a mouse model of breast cancer, we show that RANKL production by tumor-primed CD4^+^ T cells is required for development of bone metastasis. We reached this conclusion by first showing that the metastatic 4T1 tumor, but not its non-metastatic 67NR sibling, induces production of pro-osteoclastogenic cytokines, including IL-17F and RANKL by CD4^+^ T cells. Production of such cytokines leading to OC formation and activation, and osteolytic disease, is observed even before tumor cells colonize the bone cavity, suggesting that CD4^+^ T cells prepare the metastatic niche for further establishment of tumor cells in the model used. Inhibition of RANKL production by tumor-primed CD4^+^ T cells protects mice from osteolytic disease and, surprisingly, completely abolishes the development of bone metastases. Our data is in agreement with two recent studies in the transgenic MMTV-PyMT mice, a Th2 breast cancer model [14] that does not colonize the bones. In this model, metastasis to the bones are absent whereas metastasis to the lungs where shown to be Th2 dependent. However, bone metastasis did occur after a shift in the Th response from Th2 to Th17 [41,42] corroborating the need of a specific immune phenotype to allow bone colonization.

**Figure 7:**
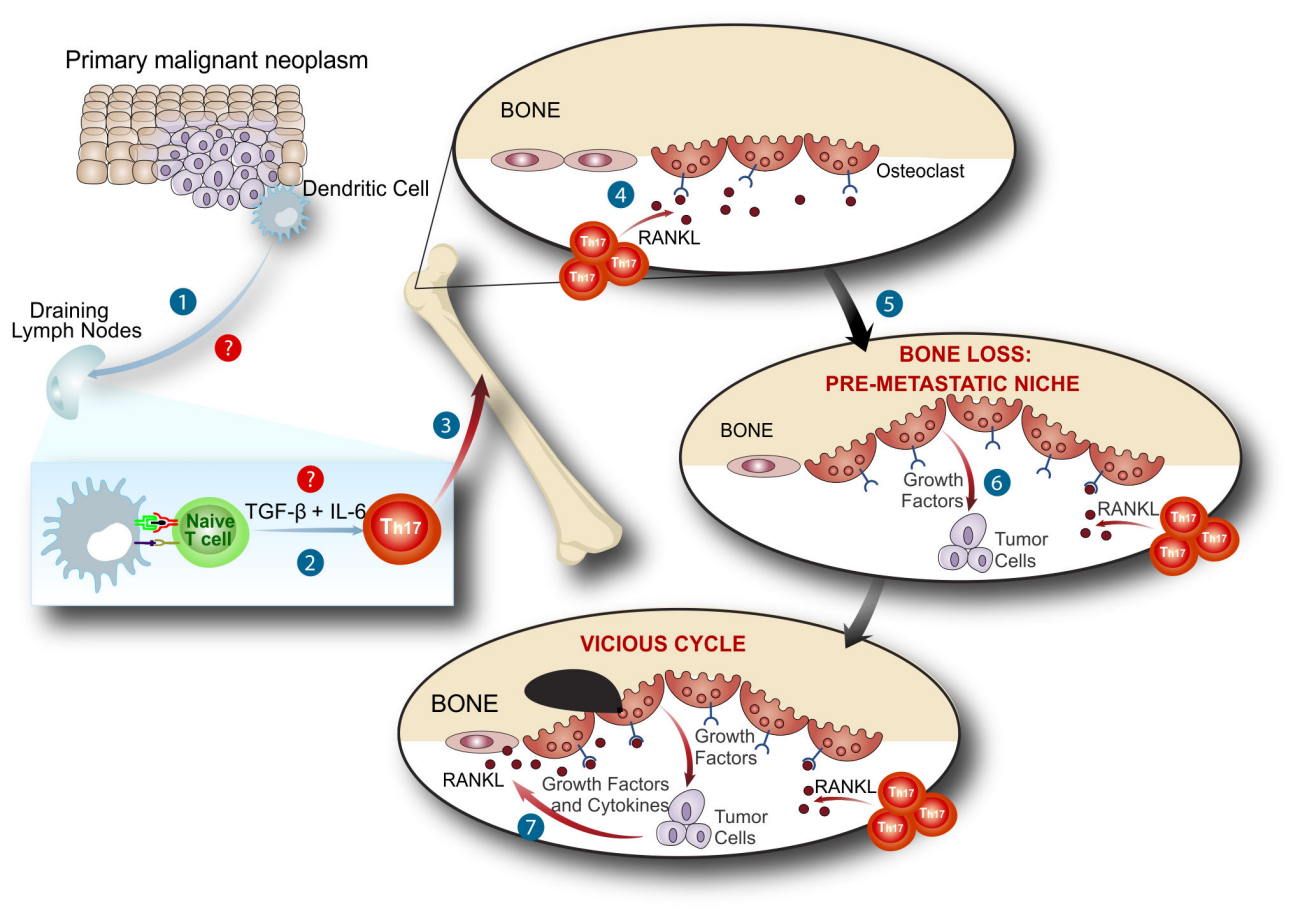
RANKL^+^ tumor-specific T cells prepare the bone pre-metastatic niche. After being stimulated (1) and modulated (2) by tumor cells T cells migrate to the bone marrow (3). When inside the bone marrow niche, T cell derived RANKL stimulate osteoclastogenesis (4) with bone consumption before tumor bone colonization (5). This initial bone loss induced by T cells in response to tumor antigen, prepares the bone marrow niche to receive tumor cells (6). Once inside the marrow, tumor cells will be able to establish themselves comfortable (7), at the expense of the pre-metastatic niche already set by T cell derived RANKL in response to tumor stimulation.

Modulation of the T cell functional phenotype to Th17 can be reached by 4T1 tumor cells but not 67NR, although either boosting with 4T1 soluble antigen extract or 67NR tumor cells has the same effect on bone loss. This suggests that the tumor antigens recognized by T cells are shared by both tumors and that the difference in the quality of the T cell response to these two sibling cell lines is probably due to differential modulation of the immune response by the tumor cells rather than being dependent on recognition of different epitopes.

The very early and intense T cell-dependent bone loss observed, in immunocompetent mice bearing 4T1 tumor or Nudes transferred with 4T1 specific T cells, is indeed surprising. The number of T cells in the BM is low, comprising 2% or less of the total marrow cell content. Also, no skew in the distribution of TCR V families was observed in the presence of 4T1 tumor arguing against any kind of superantigen stimulation by the tumor to explain the amplitude of the osteolytic bone response. These results suggest the existence of an amplifying loop triggered by the RANKL+T cell.

Contribution of T cell-derived RANKL to bone metabolism was first proposed by Penninger and colleagues in a model of inflammatory bone disease [24]. Later studies showing reversal of RANKL dependent osteopetrosis by hiperexpression of RANKL in T and B cells [43] reinforced the interplay between T cell derived RANKL and bone homeostasis. On the other hand, T cell derived IL-17A has been claimed to be pivotal to osteoclastogenesis by upregulating RANKL expression in synoviocyte and macrophages in the inflamed joint [25,30]. No direct role for IL-17 in bone physiology or cancer induced bone disease has been reported. In the 4T1 mestastatic model, IL-17F, which shows 50% homology with IL-17A and shares its receptor [44], is produced in high level but is not necessary for the development of pre-metastatic bone disease.

In a transgenic model of breast tumor with metastasis to the lungs [45], regulatory T cell-derived RANKL has been shown to be pro-metastatic. A similar role for Tregs in our model is unlikely though. First, the number of CD4^+^CD25^+^Foxp3^+^ Treg cells in mice bearing metastatic and non-metastatic tumors is the same (our unpublished results). Second, the T cell cytokine profile observed in the presence of metastatic tumor is not compatible with Treg activity. Finally, Tregs have been shown to inhibit osteoclastogenesis in other model systems [37] and in periodontal disease, Treg infiltrate is present in gingivitis preceding periodontitis. When periodontitis is established and actual bone loss takes place, Tregs disappear from the site of the lesion, giving place to RANKL^+^ and IL-17^+^ T cells [44]. Altogether, these reports indicate that Tregs are not involved in cancer-induced bone disease, although they can play a role in facilitating metastasis to organs others than the bones.

Strikingly, T cell derived RANKL expression blockage inhibits the development of bone metastasis. One could argue that the effect of RANKL inhibition in bone metastasis is secondary to the effect observed in growth of the primary tumor. Indeed, direct effect of RANKL in mammary gland cells [26] has been shown as well as in tumor aggressiveness [46,47]. However, if that was the case, meaning that delayed tumor growth would be responsible for inhibition or delay of metastasis development, the prediction would be that metastasis to organs other than the bones would be also inhibited. Yet, the number of metastatic colonies in the lungs is four times higher in the absence of RANKL ^+^ T cells indicating that the overall capacity of 4T1 tumor to produce metastasis is not impaired in the absence of T cell-derived RANKL. On the contrary, the use of osteoclast inhibitors such as anti-RANKL can certainly protect the bones but might increase the risk of pulmonary metastasis when acting over T cells, a point that needs further investigation.

We believe that the characterization of T cell-induced pre-metastatic osteolytic disease adds an extra step to the vicious cycle hypothesis (Figure 7). Tumor cells are believed to establish themselves in the BM through mechanisms that culminate in the release of growth factors from the bone matrix as a consequence of osteoclast activity. Here, we suggest that in the presence of metastatic tumors, antigen-specific T cells are primed and acquire a pro-osteoclastogenenic phenotype. Following their migratory pattern, tumor-specific primed T cells expressing RANKL migrate to the bone cavity, before tumor cells colonize it, and once there they stimulate the differentiation and activation of OCs. Pre-metastatic T cell mediated bone consumption generates a rich environment that will allow the colonization of the bone cavity by the metastatic clones. Once initial seeding of the bone tissue is achieved, the tumor cells can continue the osteolytic process on their own, feeding themselves through the vicious cycle established with the bone microenvironment.

Altogether, our results unveil an uncommon perspective of tissue-specific immune activation leading to progression of cancer and identify T cells as a major player in pre-metastatic osteolytic disease and development of bone metastasis.

## Supporting Information

Figure S1 Anti-tumor specific cytokine profile in BM cells differs between metastatic 4T1 and non-metastatic 67NR-bearing mice. BALB/c female mice were subcutaneously injected with 10^4^ 67NR or 4T1 tumor cells into the mammary fat pad. (a) Functional profile of BM tumor specific T cells among different bone marrows. 10^6^ cells from draining LNs (inguinal) and BMs, from different bones (calvariae, iliac, humerus, femur and tibiae) were stimulated in the presence or in the absence of 4T1 soluble tumor-Ag (sAg) as the antigen source. Culture supernatants were collected after 72 hs and cytokine levels were quantified by ELISA. All data are presented as the level of cytokine measured per number of CD3^+^ T cells. (b) At the indicated time points, LNs and different bones were harvested and the number of metastatic clones was determined by the 6-thioguanine resistant metastatic clonogenic assay. All data are from at least three independent experiments (n=5/mice per group) and presented as mean ± SD.
(PDF)

Figure S2 Absence of TCR Vb skew in response to tumor cells. BALB/c mice were orthotopically injected in mammary fat pad (sc.) with 10^4^ metastatic 4T1 or non-metastatic 67NR tumor cells. (a) At the indicated time points, the frequency (%) of CD3+, CD3+ CD4+ and CD3+ CD8+ T cells in LNs and iliac BMs were assessed by flow cytometry, after tumor cells injection. LN and iliac BM cells from naïve animals were used as experimental controls. (b) The absolute number of CD3+, CD3+ CD4+ and CD3+ CD8+ T cells in LNs and iliac BMs were also calculated. Data are expressed as the mean ± SD of five mice/group and are representative of at least two independent experiments. *p≤0.05; **p≤0.001. (c) TCR Vβ family distribution in the CD3+ CD4+ T cell population recovered from the draining lymph nodes or bone marrow of 4T1 recipient mice or naïve controls, 14 d p.i.
(PDF)

Figure S3 Pro-osteoclastogenic 4T1 tumor-specific T cells induce bone loss *in vivo* in the absence of tumor cells 6 days after adoptive transfer. 4T1 LN T cells were isolated from BALB/c female mice, 11 d after 4T1 tumor cells injection into the mammary fat pad. LN cells were intravenously transferred to BALB/c nude female mice along with 4T1 sAg. High resolution µCT analysis of iliac bones from nude mice, at different time points after transference of 4T1 LN T cells. The parameters calculated from µCT images were BV/TV%, trabecular bone volume/tissue volume were; total bone mineral density (g/cm^2^); trabecular number (1/mm) and trabecular thickness (mm). Values are mean ± SD of 3 mice. ^*^ *p ≤ 0.05*.
(PDF)

Figure S4 Pro-osteoclastogenic 4T1 tumor-specific T cells keep their phenotype *in vivo* after adoptive transfer. T cells were isolated from draining lymph node of BALB/c female mice, 11 d after 67NR or 4T1 tumor cells injection into mammary gland. LN cells were intravenously transferred to BALB/c nude female mice. On the same day, the animals received 67NR non-metastatic tumor cells subcutaneously as the source of Ag. T cells from naïve mice were used as controls. 14 d after transference, spleen cells were stimulated with sAg and IL-17 F and RANKL expression were either evaluated by ELISA (a) or (b) FACS. IL-17F^+^ RANKL^+^ T cells were gated on CD3^+^CD4^+^ and CD3^+^CD8^+^. (c) Sera OPG/RANKL ratio, measured by ELISA, of BALB/c mice 14 d after transference. ^*^ *p<0.05,* ^**^ *p<0.001*. (d) Bone histomorphometrical analysis of iliacs from the different experimental and control groups. Trabecular bone volume was expressed as a percentage of total tissue volume. All data are from two independent experiments (n=3/mice per group) and presented as mean ± SD. ^*^ *p≤0*.*05*.
(PDF)

Figure S5 osteolytic disease induced by T cell derived RANKL. (A) Representative micrography of the TRAP staining in the bone sections obtained in each experimental group is shown. Arrows indicate osteoclasts: 4T1 T cells (T 4T1), Naïve T cells (T Nv), no T cells (No T) or no T cell nor sAg (no T/sAg). ^*^ *p*≤0.05. (B) High resolution µCT and (C) Histomorphometric analysis of iliac bones from the different groups of nude mice transferred with the indicated T cells. Results shown are representative of two experiments with 5 mice/group). ^*^ *p*≤0.05; ^**^ *p≤0*.*001*.
(PDF)

Figure S6 Effect of T cell derived RANKL in primary tumor growth and lung meetastasisi. (A) Maximum diameter of primary tumors was determined by ultrasonography on days 12 and 26. (B) Number of metastatic clones in the lungs was assessed by clonogenic metastatic assay in the recipient mice on day 12 and 28. Nude, non-reconstituted control; T 4T1; reconstitution with 4T1 T cells; sh scr, sh Scramble T 4T1; sh RANKL, sh RANKLT 4T1. Results shown are representative of two experiments with 6 mice/group). ^**^ *p≤0*.*001*.
(PDF)
