# Supplementary figures and images for: T Cells Induce Pre-Metastatic Osteolytic Disease and Help Bone Metastases Establishment in a Mouse Model of Metastatic Breast Cancer
Source: PLoS One. 2013 Jul 18;8(7):e68171. doi: 10.1371/journal.pone.0068171 (PMC3730734; doi:10.1371/journal.pone.0068171)

Figure S1

A

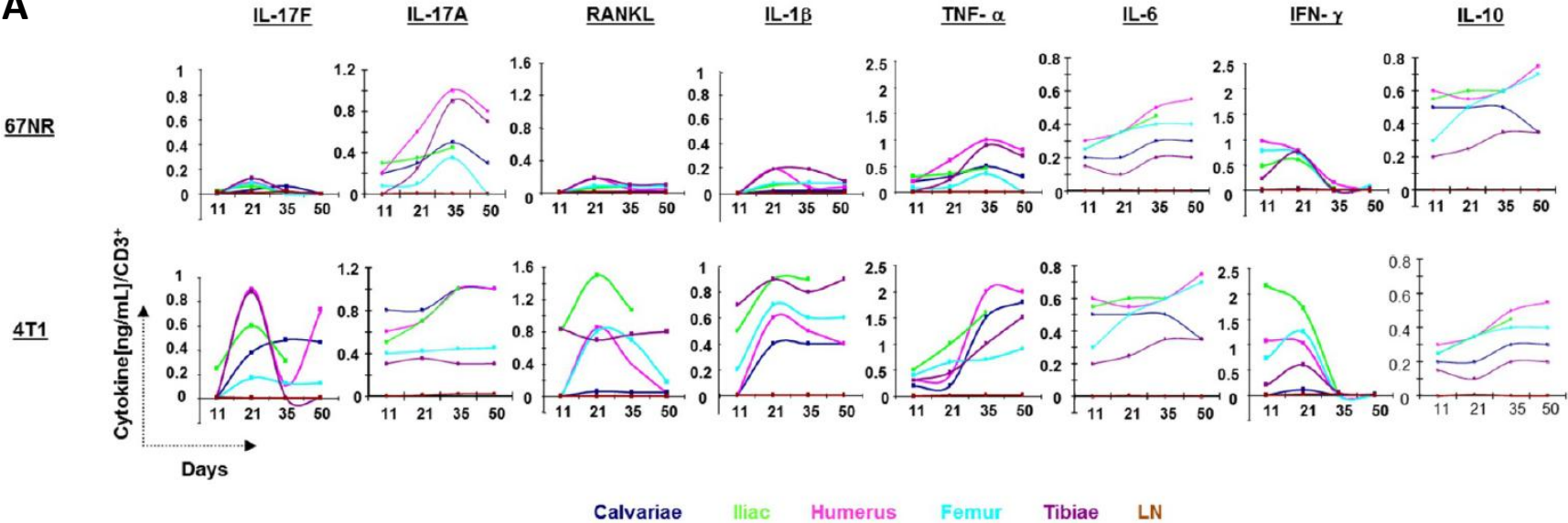

B

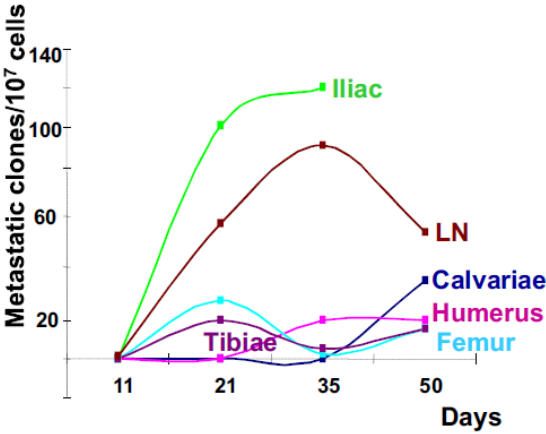

Supplement: Figure S1 — BALB/c female mice were subcutaneously injected with 104 67NR or 4T1 tumor cells into the mammary fat pad. (a) Functional profile of BM tumor specific T cells among different bone marrows. 106 cells from draining LNs (inguinal) and BMs, from different bones (calvariae, iliac, humerus, femur and tibiae) were stimulated in the presence or in the absence of 4T1 soluble tumor-Ag (sAg) as the antigen source. Culture supernatants were collected after 72 hs and cytokine levels were quantified by ELISA. All data are presented as the level of cytokine measured per number of CD3+ T cells. (b) At the indicated time points, LNs and different bones were harvested and the number of metastatic clones was determined by the 6-thioguanine resistant metastatic clonogenic assay. All data are from at least three independent experiments (n=5/mice per group) and presented as mean ± SD. (PDF) [file pone.0068171.s001.pdf]

Figure S2

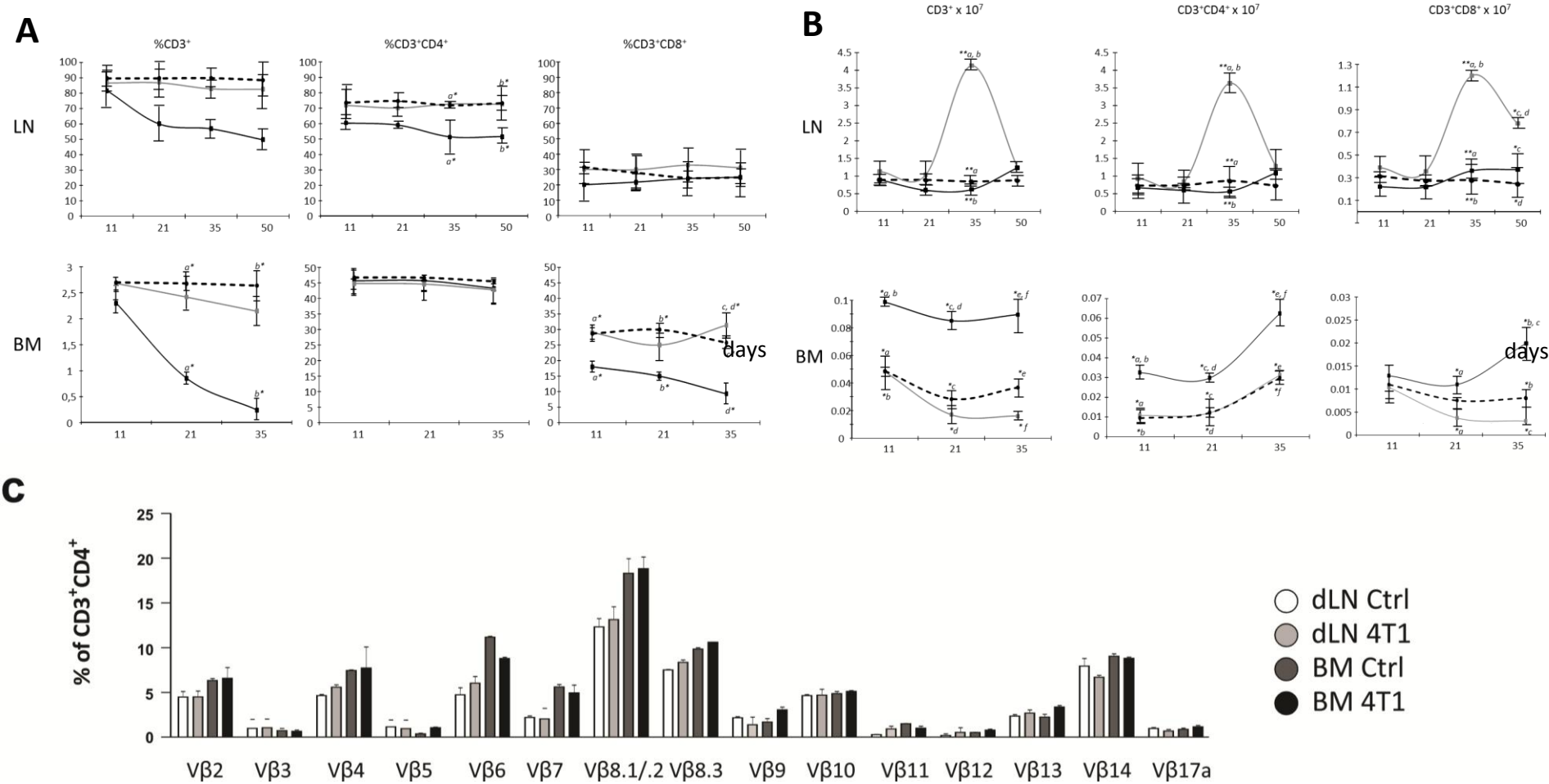

Supplement: Figure S2 — BALB/c mice were orthotopically injected in mammary fat pad (sc.) with 104 metastatic 4T1 or non-metastatic 67NR tumor cells. (a) At the indicated time points, the frequency (%) of CD3+, CD3+ CD4+ and CD3+ CD8+ T cells in LNs and iliac BMs were assessed by flow cytometry, after tumor cells injection. LN and iliac BM cells from naïve animals were used as experimental controls. (b) The absolute number of CD3+, CD3+ CD4+ and CD3+ CD8+ T cells in LNs and iliac BMs were also calculated. Data are expressed as the mean ± SD of five mice/group and are representative of at least two independent experiments. *p≤0.05; **p≤0.001. (c) TCR Vβ family distribution in the CD3+ CD4+ T cell population recovered from the draining lymph nodes or bone marrow of 4T1 recipient mice or naïve controls, 14 d p.i. (PDF) [file pone.0068171.s002.pdf]

Figure S3

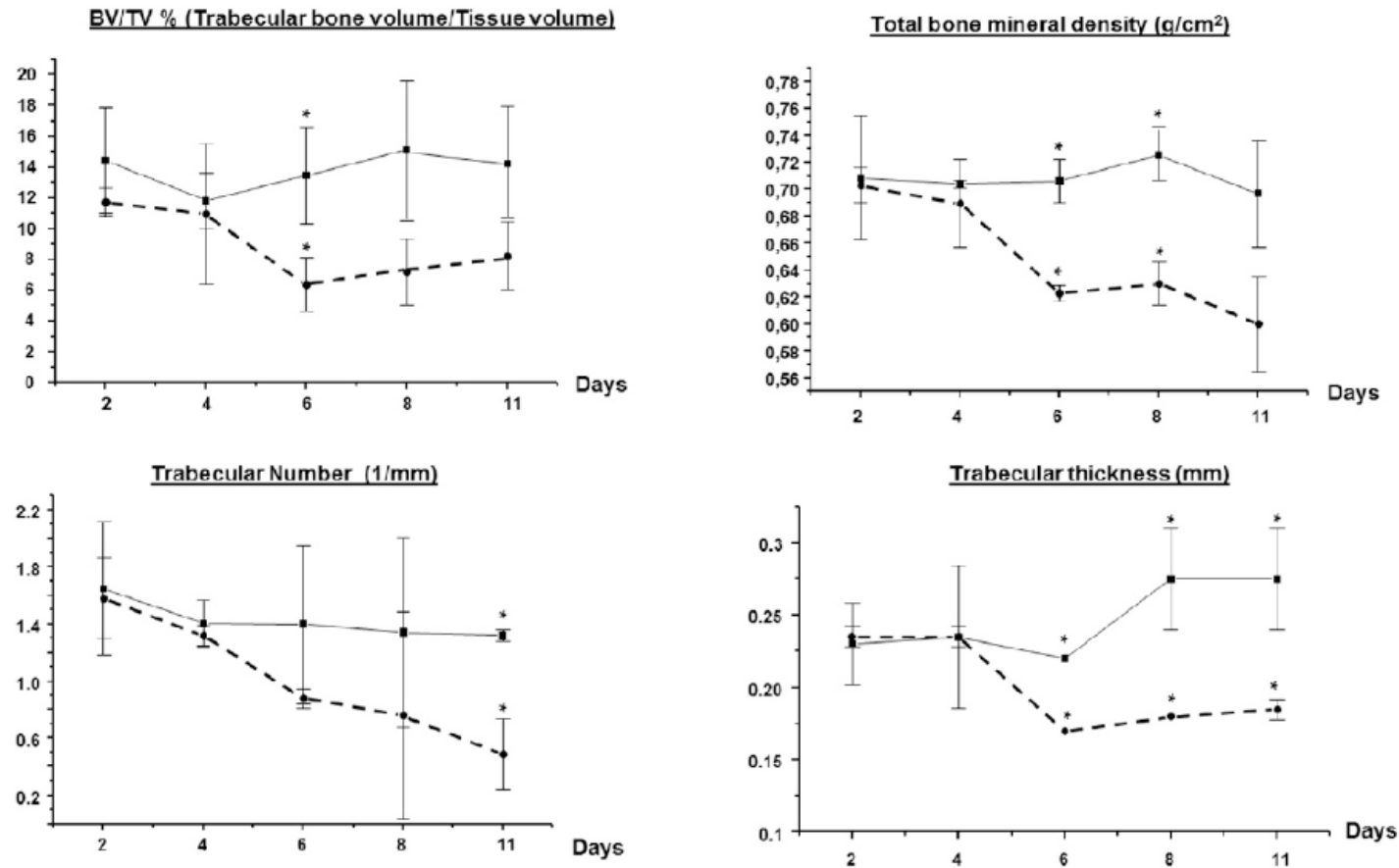

Supplement: Figure S3 — 4T1 LN T cells were isolated from BALB/c female mice, 11 d after 4T1 tumor cells injection into the mammary fat pad. LN cells were intravenously transferred to BALB/c nude female mice along with 4T1 sAg. High resolution µCT analysis of iliac bones from nude mice, at different time points after transference of 4T1 LN T cells. The parameters calculated from µCT images were BV/TV%, trabecular bone volume/tissue volume were; total bone mineral density (g/cm2); trabecular number (1/mm) and trabecular thickness (mm). Values are mean ± SD of 3 mice. * p ≤ 0.05. (PDF) [file pone.0068171.s003.pdf]

Figure S4

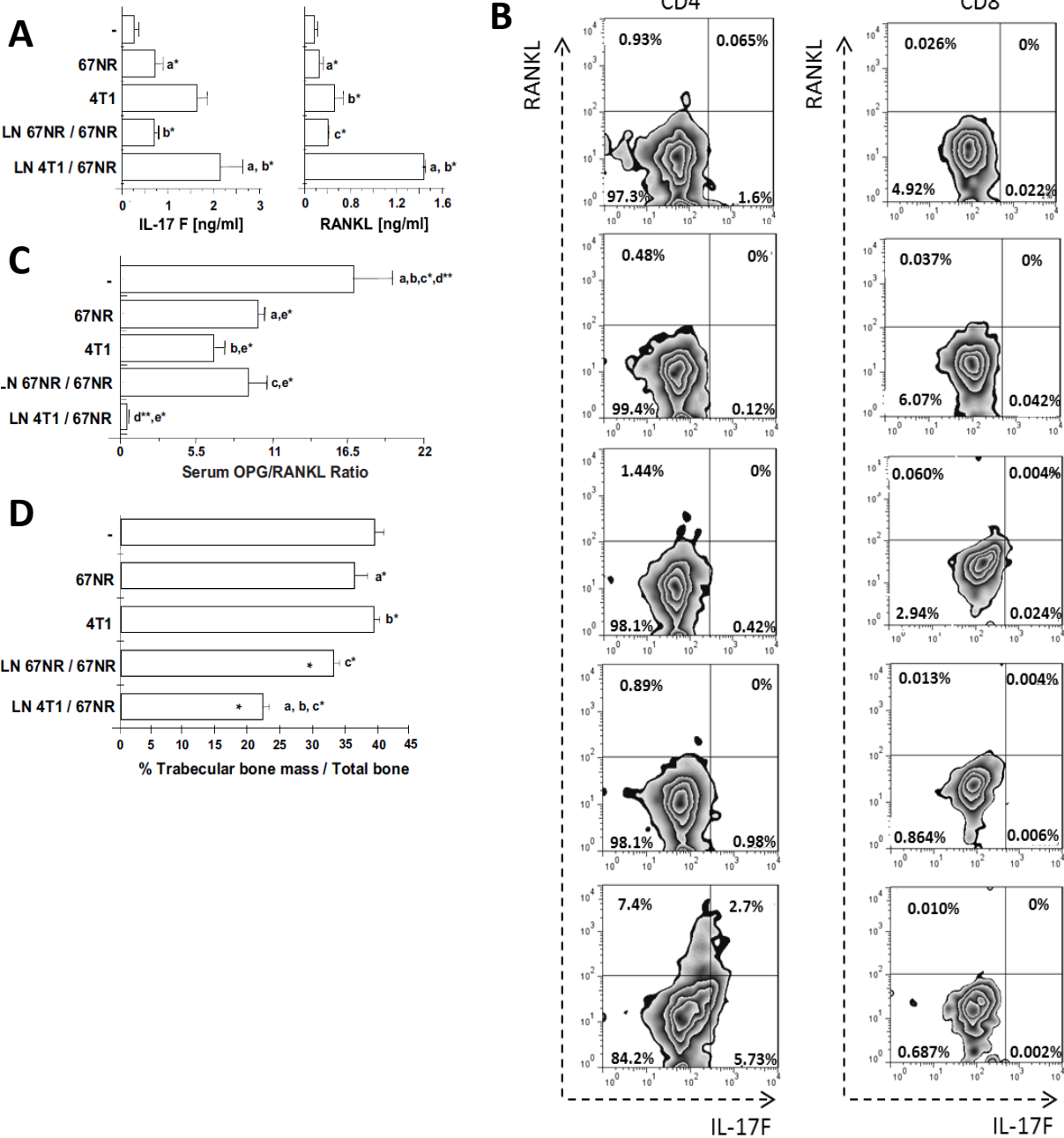

Supplement: Figure S4 — T cells were isolated from draining lymph node of BALB/c female mice, 11 d after 67NR or 4T1 tumor cells injection into mammary gland. LN cells were intravenously transferred to BALB/c nude female mice. On the same day, the animals received 67NR non-metastatic tumor cells subcutaneously as the source of Ag. T cells from naïve mice were used as controls. 14 d after transference, spleen cells were stimulated with sAg and IL-17 F and RANKL expression were either evaluated by ELISA (a) or (b) FACS. IL-17F+ RANKL+ T cells were gated on CD3+CD4+ and CD3+CD8+. (c) Sera OPG/RANKL ratio, measured by ELISA, of BALB/c mice 14 d after transference. * p<0.05, ** p<0.001. (d) Bone histomorphometrical analysis of iliacs from the different experimental and control groups. Trabecular bone volume was expressed as a percentage of total tissue volume. All data are from two independent experiments (n=3/mice per group) and presented as mean ± SD. * p≤0.05. (PDF) [file pone.0068171.s004.pdf]

Figure S5

**A**

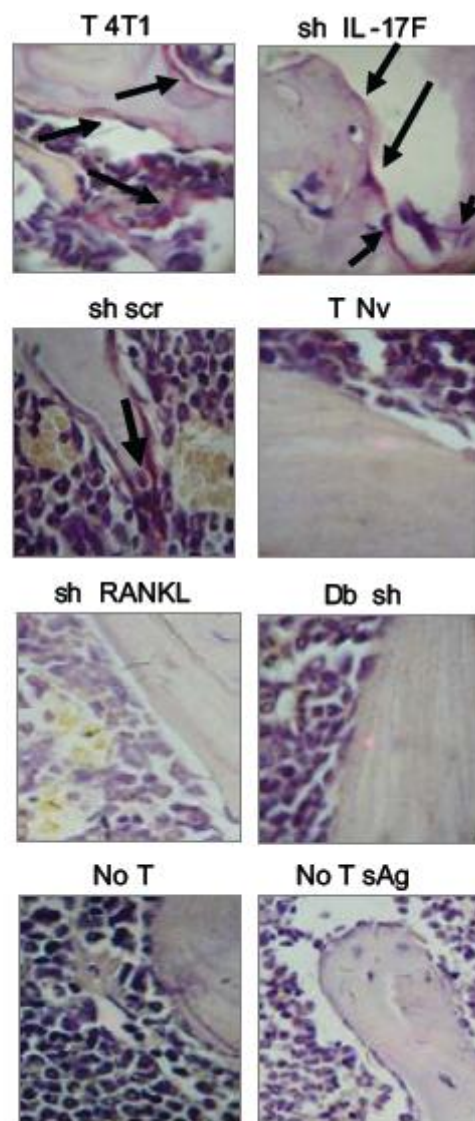

**B**

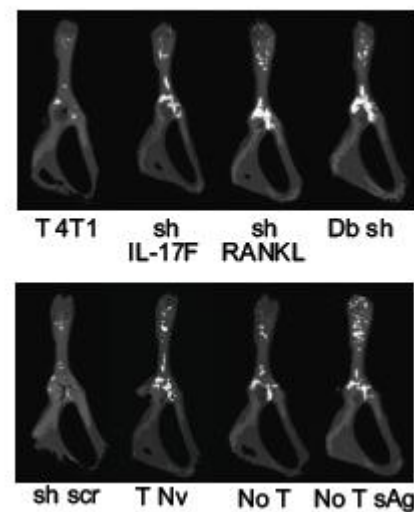

**C**

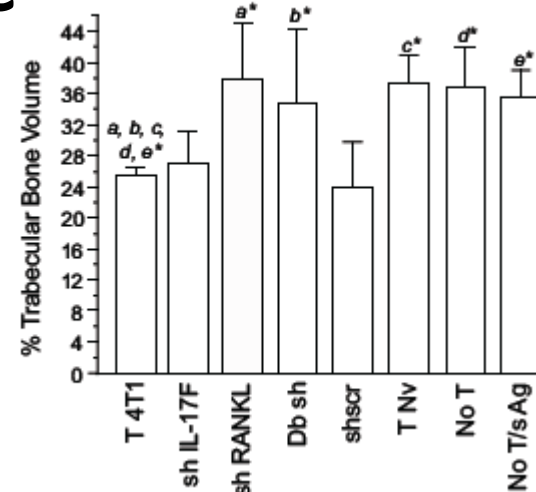

Supplement: Figure S5 — (A) Representative micrography of the TRAP staining in the bone sections obtained in each experimental group is shown. Arrows indicate osteoclasts: 4T1 T cells (T 4T1), Naïve T cells (T Nv), no T cells (No T) or no T cell nor sAg (no T/sAg). * p≤0.05. (B) High resolution µCT and (C) Histomorphometric analysis of iliac bones from the different groups of nude mice transferred with the indicated T cells. Results shown are representative of two experiments with 5 mice/group). * p≤0.05; ** p≤0.001. (PDF) [file pone.0068171.s005.pdf]

**A**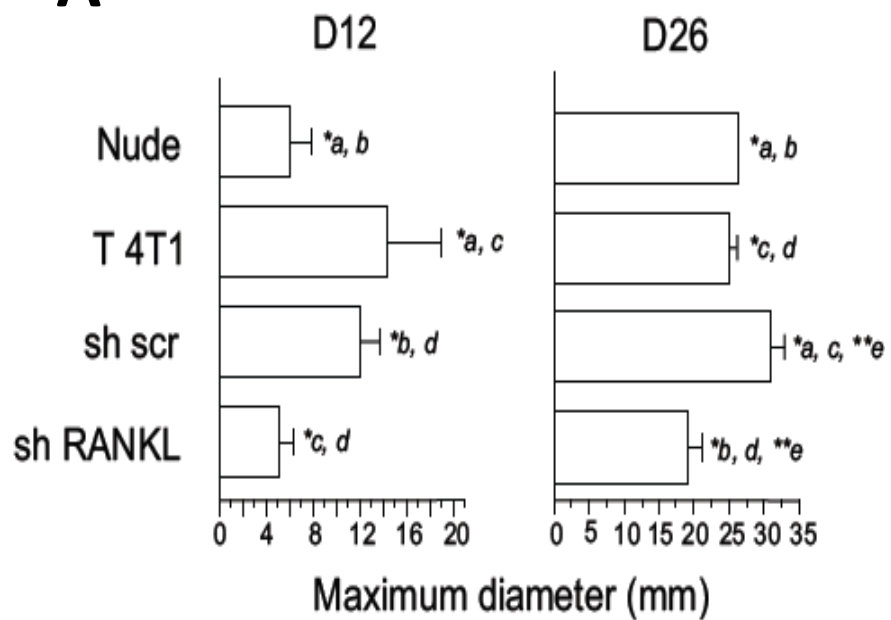**B**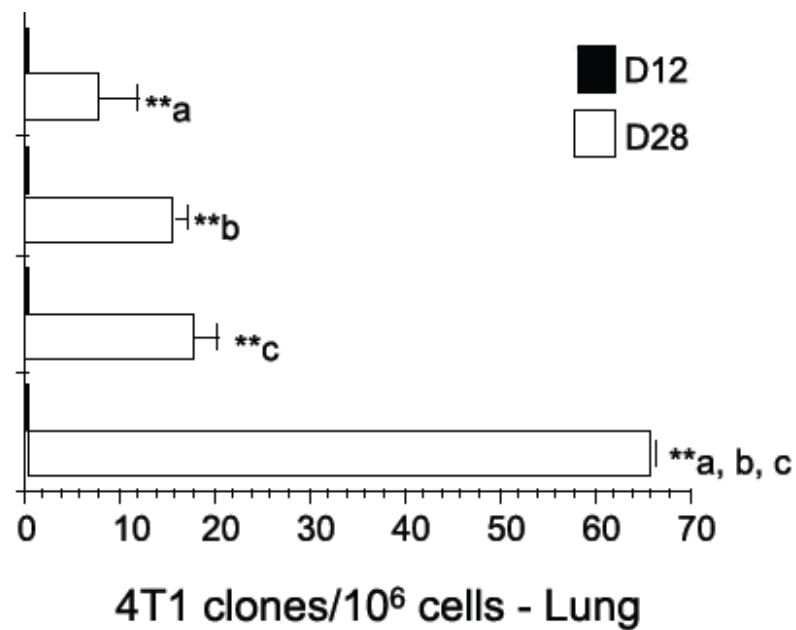

Supplement: Figure S6 — (A) Maximum diameter of primary tumors was determined by ultrasonography on days 12 and 26. (B) Number of metastatic clones in the lungs was assessed by clonogenic metastatic assay in the recipient mice on day 12 and 28. Nude, non-reconstituted control; T 4T1; reconstitution with 4T1 T cells; sh scr, sh Scramble T 4T1; sh RANKL, sh RANKLT 4T1. Results shown are representative of two experiments with 6 mice/group). ** p≤0.001. (PDF) [file pone.0068171.s006.pdf]
